# 5-Bromo­phthalazine hemihydrate

**DOI:** 10.1107/S1600536812030358

**Published:** 2012-07-10

**Authors:** Mingjian Cai

**Affiliations:** a Department of Chemistry, Tangshan Normal University, Tangshan 063000, People’s Republic of China

## Abstract

The title compound, C_8_H_5_BrN_2_·0.5H_2_O, is a phthalazine derivative synthesized from 3-bromo­benzene-1,2-dicarbaldehyde and hydrazine. The mol­ecule is essentially planar, the deviation from the mean plane of the phthalazine ring being 0.015 (3) Å. The O atom of the solvent water mol­ecule is situated on a twofold rotation axis. In the crystal, O—H⋯N hydrogen bonds and short N⋯Br [2.980 (3) Å] contacts lead to the formation of a two-dimensional network parallel to (101).

## Related literature

For general background on applications of phthalazines, see: Caira *et al.* (2011 ▶); Musa *et al.* (2012 ▶).
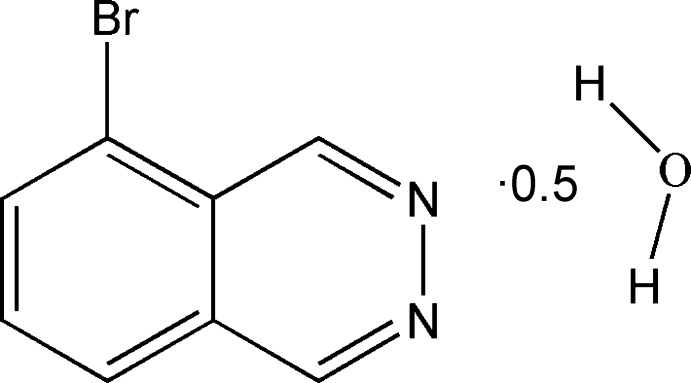


## Experimental

### 

#### Crystal data

C_8_H_5_BrN_2_·0.5H_2_O
*M* *_r_* = 218.06
Orthorhombic, 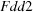

*a* = 13.5000 (18) Å
*b* = 29.964 (5) Å
*c* = 7.5565 (5) Å
*V* = 3056.7 (7) Å^3^
*Z* = 16
Mo *K*α radiation
μ = 5.31 mm^−1^
*T* = 113 K
0.30 × 0.22 × 0.18 mm

#### Data collection

Rigaku Saturn724 CCD diffractometer
Absorption correction: multi-scan (*CrystalClear*; Rigaku/MSC, 2002 ▶) *T*_min_ = 0.299, *T*_max_ = 0.448
7799 measured reflections
1819 independent reflections
1781 reflections with *I* > 2σ(*I*)
*R*_int_ = 0.064

#### Refinement

*R*[*F* ^2^ > 2σ(*F* ^2^)] = 0.029
*wR*(*F* ^2^) = 0.074
*S* = 1.06
1819 reflections
109 parameters
2 restraints
H atoms treated by a mixture of independent and constrained refinement
Δρ_max_ = 0.74 e Å^−3^
Δρ_min_ = −0.77 e Å^−3^
Absolute structure: Flack (1983 ▶), 839 Friedel pairs
Flack parameter: −0.002 (10)

### 

Data collection: *CrystalClear* (Rigaku/MSC, 2002 ▶); cell refinement: *CrystalClear*; data reduction: *CrystalClear*; program(s) used to solve structure: *SHELXS97* (Sheldrick, 2008 ▶); program(s) used to refine structure: *SHELXL97* (Sheldrick, 2008 ▶); molecular graphics: *DIAMOND* (Crystal Impact, 2009 ▶); software used to prepare material for publication: *CrystalStructure* (Rigaku/MSC, 2006 ▶).

## Supplementary Material

Crystal structure: contains datablock(s) I, global. DOI: 10.1107/S1600536812030358/im2382sup1.cif

Structure factors: contains datablock(s) I. DOI: 10.1107/S1600536812030358/im2382Isup2.hkl

Supplementary material file. DOI: 10.1107/S1600536812030358/im2382Isup3.cml

Additional supplementary materials:  crystallographic information; 3D view; checkCIF report

## Figures and Tables

**Table 1:** Hydrogen-bond geometry (Å, °)

*D*—H⋯*A*	*D*—H	H⋯*A*	*D*⋯*A*	*D*—H⋯*A*
O1—H1*A*⋯N1	0.82 (2)	2.07 (3)	2.887 (3)	175 (5)

**Fig. 1.**
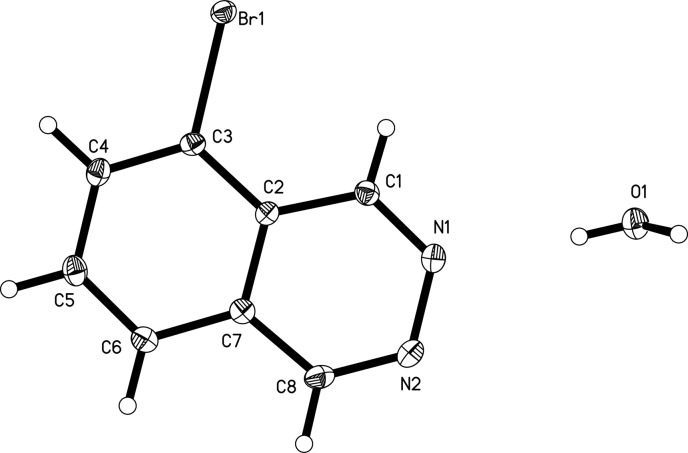
Molecular structure of the title compound showing 30% probability displacement ellipsoids.

**Fig. 2.**
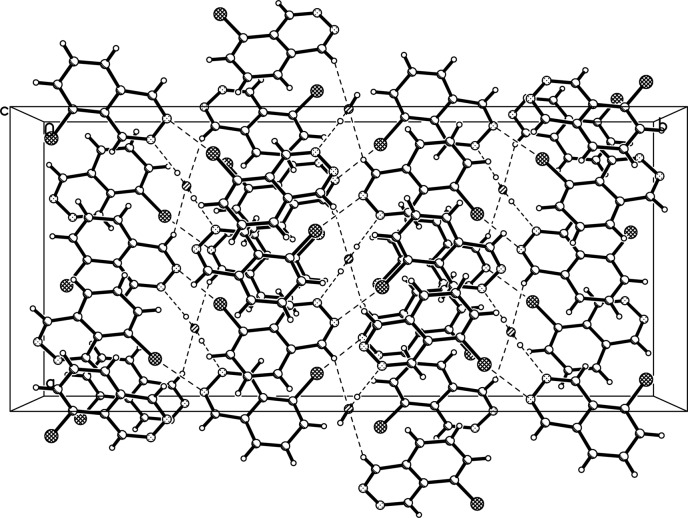
Molecular packing of the title compound with hydrogen bonds and N···Br contacts shown as dashed lines.

## supplementary crystallographic information

### Comment

Phthalazine derivatives have played an important role in the development of corrosion science as they can inhibit the corrosion of mild steel (Musa *et al.*, 2012). Moreover, they are of particular interest owing to their biological activity and optical properties (Caira, *et al.*, 2011).

In this paper, the title new phthalazine derivative derived from the condensation of 3-bromo-benzene-1,2-dicarboxaldehyde with hydrazine is reported. The molecular structure of the title compound (Fig.1) is essentially planar with a deviation from the mean plane of the phthalazine ring of 0.0115 (3) Å. All bond lengths have normal values.The oxygen atom of the solvent water molecule is situated on a twofold rotation axis. In the crystal, O—H···N hydrogen bonds and short N···Br contacts lead to the formation of a two dimensional network structure (Fig.2).

### Experimental

A solution of 0.1 mol of 3-bromo-benzene-1,2-dicarboxaldehyde is dissolved in 100 ml of ethanol and added dropwise with constant stirring, under a blanket of nitrogen, to an ice-cooled solution of 0.3 mol of hydrazine hydrate in 100 ml of ethanol. The light yellowish reaction mixture is kept with constant stirring for an additional three hours. Ethanol and excess hydrazine are removed under reduced pressure. The remaining yellowish solid may be purified by recrystallization from diethyl ether to yield the yellowish title compound (yield 48%). Finally, the title compound was dissolved in a small amount of methanol and the solution was kept for 10 days at ambient temperature to give rise to white flake crystals due to slow evaporation of the solvent.

### Refinement

The H atom of the solvent water was located in a difference fourier map and refined as a riding atom with *U*_iso_(H) = 1.2*U*_eq_(O). Remaining H atoms were positioned geometrically with C—H = 0.93–0.98 Å and constrained to ride on their parent atoms with *U*_iso_(H) = 1.2*U*_eq_(C).

### Crystal data

**Table:**

C_8_H_5_BrN_2_·0.5H_2_O	*F*(000) = 1712
*M**_r_* = 218.06	*D*_x_ = 1.895 Mg m^−^^3^
Orthorhombic, *F**d**d*2	Mo *K*α radiation, λ = 0.71073 Å
Hall symbol: F 2 -2d	Cell parameters from 2874 reflections
*a* = 13.5000 (18) Å	θ = 1.4–28.0°
*b* = 29.964 (5) Å	µ = 5.31 mm^−^^1^
*c* = 7.5565 (5) Å	*T* = 113 K
*V* = 3056.7 (7) Å^3^	Prism, colorless
*Z* = 16	0.30 × 0.22 × 0.18 mm

### Data collection

**Table:**

Rigaku Saturn724 CCD diffractometer	1819 independent reflections
Radiation source: rotating anode	1781 reflections with *I* > 2σ(*I*)
Multilayer monochromator	*R*_int_ = 0.064
Detector resolution: 14.22 pixels mm^-1^	θ_max_ = 27.8°, θ_min_ = 2.7°
ω and φ scans	*h* = −17→17
Absorption correction: multi-scan (*CrystalClear*; Rigaku/MSC, 2002)	*k* = −37→39
*T*_min_ = 0.299, *T*_max_ = 0.448	*l* = −9→9
7799 measured reflections	

### Refinement

**Table:**

Refinement on *F*^2^	Secondary atom site location: difference Fourier map
Least-squares matrix: full	Hydrogen site location: inferred from neighbouring sites
*R*[*F*^2^ > 2σ(*F*^2^)] = 0.029	H atoms treated by a mixture of independent and constrained refinement
*wR*(*F*^2^) = 0.074	*w* = 1/[σ^2^(*F*_o_^2^) + (0.0496*P*)^2^] where *P* = (*F*_o_^2^ + 2*F*_c_^2^)/3
*S* = 1.06	(Δ/σ)_max_ = 0.001
1819 reflections	Δρ_max_ = 0.74 e Å^−^^3^
109 parameters	Δρ_min_ = −0.77 e Å^−^^3^
2 restraints	Absolute structure: Flack (1983), 839 Friedel pairs
Primary atom site location: structure-invariant direct methods	Flack parameter: −0.002 (10)

### Special details

**Table:**

Geometry. All e.s.d.'s (except the e.s.d. in the dihedral angle between two l.s. planes) are estimated using the full covariance matrix. The cell e.s.d.'s are taken into account individually in the estimation of e.s.d.'s in distances, angles and torsion angles; correlations between e.s.d.'s in cell parameters are only used when they are defined by crystal symmetry. An approximate (isotropic) treatment of cell e.s.d.'s is used for estimating e.s.d.'s involving l.s. planes.
Refinement. Refinement of *F*^2^ against ALL reflections. The weighted *R*-factor *wR* and goodness of fit *S* are based on *F*^2^, conventional *R*-factors *R* are based on *F*, with *F* set to zero for negative *F*^2^. The threshold expression of *F*^2^ > σ(*F*^2^) is used only for calculating *R*-factors(gt)*etc*. and is not relevant to the choice of reflections for refinement. *R*-factors based on *F*^2^ are statistically about twice as large as those based on *F*, and *R*-factors based on ALL data will be even larger.

### Fractional atomic coordinates and isotropic or equivalent isotropic displacement parameters (Å^2^)

**Table:**

	*x*	*y*	*z*	*U*_iso_*/*U*_eq_	
Br1	0.655288 (19)	0.300585 (8)	0.53761 (6)	0.02108 (10)	
N1	0.65121 (19)	0.45112 (8)	0.5432 (5)	0.0234 (5)	
N2	0.71705 (19)	0.47790 (9)	0.6304 (4)	0.0256 (6)	
C1	0.6599 (2)	0.40781 (10)	0.5487 (6)	0.0204 (6)	
H1	0.6127	0.3904	0.4862	0.024*	
C2	0.7360 (2)	0.38513 (10)	0.6426 (4)	0.0176 (5)	
C3	0.7467 (2)	0.33818 (10)	0.6536 (4)	0.0184 (6)	
C4	0.8227 (2)	0.32011 (10)	0.7484 (4)	0.0220 (6)	
H4	0.8286	0.2886	0.7578	0.026*	
C5	0.8923 (2)	0.34786 (12)	0.8323 (4)	0.0245 (7)	
H5	0.9461	0.3348	0.8946	0.029*	
C6	0.8843 (2)	0.39313 (11)	0.8259 (4)	0.0228 (7)	
H6	0.9313	0.4115	0.8846	0.027*	
C7	0.8049 (2)	0.41233 (10)	0.7306 (4)	0.0189 (6)	
C8	0.7888 (2)	0.45888 (11)	0.7189 (5)	0.0255 (7)	
H8	0.8337	0.4778	0.7801	0.031*	
O1	0.5000	0.5000	0.3550 (5)	0.0295 (8)	
H1A	0.543 (2)	0.4874 (15)	0.413 (5)	0.045 (14)*	

### Atomic displacement parameters (Å^2^)

**Table:**

	*U*^11^	*U*^22^	*U*^33^	*U*^12^	*U*^13^	*U*^23^
Br1	0.02422 (15)	0.01764 (14)	0.02139 (15)	−0.00117 (9)	−0.00086 (12)	−0.00143 (13)
N1	0.0232 (12)	0.0237 (12)	0.0231 (13)	0.0039 (8)	0.0011 (11)	0.0018 (14)
N2	0.0291 (14)	0.0195 (12)	0.0281 (15)	0.0028 (10)	0.0021 (12)	−0.0008 (12)
C1	0.0179 (12)	0.0221 (13)	0.0211 (15)	0.0007 (10)	0.0008 (12)	−0.0016 (16)
C2	0.0202 (14)	0.0169 (13)	0.0157 (14)	0.0005 (11)	0.0021 (11)	−0.0001 (12)
C3	0.0226 (13)	0.0184 (14)	0.0141 (12)	−0.0010 (10)	0.0015 (11)	−0.0028 (11)
C4	0.0273 (14)	0.0201 (14)	0.0185 (15)	0.0043 (12)	0.0017 (12)	0.0000 (12)
C5	0.0232 (15)	0.0290 (16)	0.0214 (17)	0.0063 (13)	0.0003 (11)	0.0005 (12)
C6	0.0212 (14)	0.0255 (14)	0.0215 (18)	−0.0005 (12)	−0.0004 (11)	−0.0018 (12)
C7	0.0193 (13)	0.0220 (14)	0.0154 (13)	0.0011 (11)	0.0036 (10)	−0.0018 (11)
C8	0.0265 (16)	0.0216 (14)	0.0284 (16)	−0.0023 (11)	0.0020 (13)	−0.0067 (13)
O1	0.0263 (18)	0.0323 (18)	0.0298 (18)	0.0044 (14)	0.000	0.000

### Geometric parameters (Å, º)

**Table:**

Br1—C3	1.887 (3)	C4—C5	1.406 (5)
N1—C1	1.304 (4)	C4—H4	0.9500
N1—N2	1.367 (4)	C5—C6	1.362 (5)
N2—C8	1.308 (4)	C5—H5	0.9500
C1—C2	1.421 (4)	C6—C7	1.413 (4)
C1—H1	0.9500	C6—H6	0.9500
C2—C7	1.405 (4)	C7—C8	1.414 (4)
C2—C3	1.417 (4)	C8—H8	0.9500
C3—C4	1.363 (4)	O1—H1A	0.82 (2)
			
C1—N1—N2	120.7 (3)	C5—C4—H4	119.8
C8—N2—N1	118.2 (3)	C6—C5—C4	121.3 (3)
N1—C1—C2	123.9 (3)	C6—C5—H5	119.3
N1—C1—H1	118.1	C4—C5—H5	119.3
C2—C1—H1	118.1	C5—C6—C7	118.9 (3)
C7—C2—C3	118.7 (3)	C5—C6—H6	120.5
C7—C2—C1	115.9 (3)	C7—C6—H6	120.5
C3—C2—C1	125.3 (3)	C2—C7—C6	120.5 (3)
C4—C3—C2	120.2 (3)	C2—C7—C8	116.2 (3)
C4—C3—Br1	119.9 (2)	C6—C7—C8	123.3 (3)
C2—C3—Br1	119.9 (2)	N2—C8—C7	125.1 (3)
C3—C4—C5	120.3 (3)	N2—C8—H8	117.4
C3—C4—H4	119.8	C7—C8—H8	117.4
			
C1—N1—N2—C8	0.1 (5)	C4—C5—C6—C7	−1.0 (5)
N2—N1—C1—C2	0.0 (6)	C3—C2—C7—C6	1.0 (4)
N1—C1—C2—C7	−0.9 (5)	C1—C2—C7—C6	−179.1 (3)
N1—C1—C2—C3	178.9 (4)	C3—C2—C7—C8	−178.3 (3)
C7—C2—C3—C4	0.1 (4)	C1—C2—C7—C8	1.6 (4)
C1—C2—C3—C4	−179.8 (3)	C5—C6—C7—C2	−0.6 (4)
C7—C2—C3—Br1	179.8 (2)	C5—C6—C7—C8	178.7 (3)
C1—C2—C3—Br1	0.0 (4)	N1—N2—C8—C7	0.7 (5)
C2—C3—C4—C5	−1.6 (5)	C2—C7—C8—N2	−1.6 (5)
Br1—C3—C4—C5	178.7 (2)	C6—C7—C8—N2	179.1 (3)
C3—C4—C5—C6	2.0 (5)		

### Hydrogen-bond geometry (Å, º)

**Table:**

*D*—H···*A*	*D*—H	H···*A*	*D*···*A*	*D*—H···*A*
O1—H1*A*···N1	0.82 (2)	2.07 (3)	2.887 (3)	175 (5)
